# 

**Published:** 1845-03

**Authors:** 


### CONTENTS

OF NO. III.

### ORIGINAL COMMUNICATIONS.

### ESSAYS AND CASES.

Art. I.—Observations on the Botany of Illinois, more especially in reference to the Autumnal Flora of the Prairies. In a letter to Daniel Drake, M.D., &c. By C. W. Short, M.D. - - - - - - - 187

Art. II.—A Case in which a sharp-pointed body was swallowed by a child, passing the bowels without injury. By B. W. Avent, of Murfreesboro', Tenn. - - 198

### REVIEWS.

Art. III.—Medical Topography of Brazil and Uruguay: with incidental remarks. By G. R. B. Horner, M. D., Surgeon U. S. Navy; Honorary Member of the Philadelphia Medical Society, and Correspanding Member of the National Institute at Washington, - 201

Art. IV.—Elements of Materia Medica and Therapeutics. By John P. Harrison, M.D., Professor of Materia Medica and Therapeutics in the Medical College of Ohio, - - - - - - - - - - - - 219

Art. V.—An Essay on Curvatures and Diseases of the Spine, including all the forms of Spinal Distortion. By R. W. Bampfield, Esq., one of the Surgeons to the Royal Metrapolitan Infirmary for Diseases of Children, &c., &c. Edited by J. K. Mitchell, M. D., Professor of the Practice of Medicine in Jefferson College of Philadelphia, &c., &c., - - - 233

Art. VI.—A Synopsis of the Symptoms, Diagnosis, and Treatment of the more Common and Important Diseases of the Skin. With 60 colored figures. By N. Worcester, M.D., Professor of Physical Diagnosis and Pathology in the Medical School of Cleveland; late Professor in the Medical College of Ohio, 238

SELECTIONS FROM AMERICAN AND FOREIGN JOURNALS

Report of the Trial of Abner Rogers, Jr., indicted for the murder of Charles Lincoln, Jr., late Warden of the Massachusetts State Prison; before the Supreme Judicial Court of Massachusetts; holden at Boston, on Tuesday, January 30, 1844, - - - - - - 245
Some Hints on the most Efficient Modes of Administering Medicines, - - - - - - - - - 258
On the Use of Iodide of Potassium in Asthma, - - - 260
Amputation in the Prairies, - - - - - - - 262
Mesmerism, - - - - - - - - - - 263
On the Use of Arsenic in Destroying the Sensibility of a Carious Tooth, preparatory to Filling, - - - 266
Punishment of Quacks in Olden Times, - - - - 268

ORIGINAL INTELLIGENCE.

Unofficial Editorial Notice, - - - - - - 269
Study of Botany by Young Physicians, - - - - 266
Cincinnati Journal of Health, - - - - - - - - 273
Kentucky Institution for the Education of the Blind, - - 274
Cooper on the Testis, - - - - - - - - - 275
Taylor's Medical Jurisprudence, - - - - - - 276
Bloomingdale Asylum for the Insane, - - - - - 276

